# Comparison of Hybrid Capture 2 Assay with Real-time-PCR for Detection and Quantitation of Hepatitis B Virus DNA

**DOI:** 10.5005/jp-journals-10018-1093

**Published:** 2014-01-22

**Authors:** Farjana Majid, Munira Jahan, Ahmed Lutful Moben, Shahina Tabassum

**Affiliations:** 1 Department of Microbiology, Tairunnessa Memorial Medical College, Tongi, Gazipur, Bangladesh; 2 Department of Virology, Bangabandhu Sheikh Mujib Medical University, Dhaka, Bangladesh; 3 Department of Medicine, Shaheed Suhrawardy Medical College Hospital, Dhaka, Bangladesh; 4 Department of Virology, Bangabandhu Sheikh Mujib Medical University, Dhaka, Bangladesh

**Keywords:** HBV DNA, Real-time-PCR, HBeAg, HC2 assay.

## Abstract

**Background:**

Both real-time-polymerase chain reaction (PCR) and hybrid capture 2 (HC2) assay can detect and quantify hepatitis B virus (HBV) DNA. However, real-time-PCR can detect a wide range of HBV DNA, while HC2 assay could not detect lower levels of viremia. The present study was designed to detect and quantify HBV DNA by real-time-PCR and HC2 assay and compare the quantitative data of these two assays.

**Materials and methods:**

A cross-sectional study was conducted in between July 2010 and June 2011. A total of 66 serologically diagnosed chronic hepatitis B (CHB) patients were selected for the study. Real-time-PCR and HC2 assay was done to detect HBV DNA. Data were analyzed by statistical Package for the social sciences (SPSS).

**Results:**

Among 66 serologically diagnosed chronic hepatitis B patients 40 (60.61%) patients had detectable and 26 (39.39%) had undetectable HBV DNA by HC2 assay. Concordant results were obtained for 40 (60.61%) out of these 66 patients by real-time-PCR and HC2 assay with mean viral load of 7.06 ± 1.13 log_10_ copies/ml and 6.95 ± 1.08 log_10_ copies/ml, respectively. In the remaining 26 patients, HBV DNA was detectable by real-time-PCR in 20 patients (mean HBV DNA level was 3.67 ± 0.72 log_10_ copies/ml. However, HBV DNA could not be detectable in six cases by the both assays. The study showed strong correlation (r = 0.915) between real-time-PCR and HC2 assay for the detection and quantification of HBV DNA.

**Conclusion:**

HC2 assay may be used as an alternative to real-time-PCR for CHB patients.

**How to cite this article:** Majid F, Jahan M, Moben AL, Tabassum S. Comparison of Hybrid Capture 2 Assay with Real-time-PCR for Detection and Quantitation of Hepatitis B Virus DNA. Euroasian J Hepato-Gastroenterol 2014;4(1):31-35.

## INTRODUCTION

Bangladesh is a densely populated country with intermediate endemicity for chronic hepatitis B (CHB) infection.^1^ Studies have shown that hepatitis B virus (HBV) is responsible for 31.25% cases of acute hepatitis, 76.3% cases of chronic hepatitis, 61.15% cases of cirrhosis of liver and 33.3% cases of hepatocellular carcinoma in Bangladesh.^2-4^ It was thought that seroconversion from HBeAg to HBeAb is accompanied by cessation of HBV replication and remission of liver disease. But, in the last couple of years, HBeAg-negative CHB is recognized as an important form of chronic hepatitis, where HBeAg negativity is due to mutations in precore and core promoter regions.^5^

Viral load tests that quantify HBV in peripheral blood are currently the most useful and most widely used. High-sensitivity molecular assays are clearly important for the diagnosis of HBeAg negative CHB and occult HBV, where viral loads can be quite low.^6,7^

HBV hybrid capture 2 (HC2) assay is a liquid hybridization assay based on signal amplification and can detect 4.0 × 10^5^ copies/ml.^5^ HBV HC2 assay offer high specificity to detect HBV DNA. It is able to quantify HBV DNA between 1.4 × 10^5^ and 1.7 × 10^9^ HBV copies per ml in a standard format.^8^ Signal amplification-based assays are less sensitive and may not allow the detection of early elevations in viral loads until they reach 10^5^ HBV DNA copies per ml.^9^ Standard antiviral treatment with nucleoside analogs can reduce HBV DNA levels within a few weeks to a level not detectable by these assays.^10^

The recent introduction of fluorescence resonance energy transfer (FRET)-based real-time polymerase chain reaction (PCR) has been particularly advantageous for HBV DNA quantification, because it provides high sensitivity with a much broader dynamic range than alternative assay types.^11-14^ Since amplification, measurement and quantification of PCR product occur simultaneously in the same closed reaction vessel, the need for post-PCR manipulations is obviated and the risk of PCR product carryover contamination is minimized. Several studies have compared between real-time-PCR and HC2 assay for detection of HBV DNA to determine its usefulness for a clinical virology laboratory. No difference in detection of HBV DNA between the HC2 assay and real-time-PCR assay was determined within the range of the HC2 assay.^15-17^

Several studies from Bangladesh have shown that the level of HBV DNA in serum or plasma correlate with biochemical and histological measures of disease and probably reflects the replicative activity of HBV more accurately. However, there are no studies on comparison of HBV DNA level by real-time-PCR and HC2 assay.

## MATERIALS AND METHODS

This cross-sectional study was carried out among chronic HBV-infected patient during the period of July 2010 to June 2011. Collection of specimens and laboratory work was carried out at the Department of Virology, Bangabandhu Sheikh Mujib Medical University (BSMMU). The study population consisted of 66 serologically diagnosed CHB patients. The results of these 66 samples were compared by both real-time-PCR and HC2 assay for HBV DNA detection and quantification. The history of patients was recorded in predesigned data collection sheet.

The HBV DNA was quantified with a commercially available kit (RoboGene HBV DNA Quantification Kit, Lot no- 009, Germany) and (Digene Corp, Lot no-5274625, Gaithersburg, MD, USA) according to the manufacturer’s instructions.

Results were expressed as mean ± standard deviation (SD) or percentage. Statistical analysis of HBV DNA value was performed after log_10_ conversion. Fisher’s exact test and Mann-Whitney U test were used for comparison. Pearson correlation was performed for correlation analysis. Statistical analysis was made using statistical package for the social sciences (SPSS) 17.0 software and p-value of <0.05 considered as significant.

## RESULTS

**Fig. 1:**
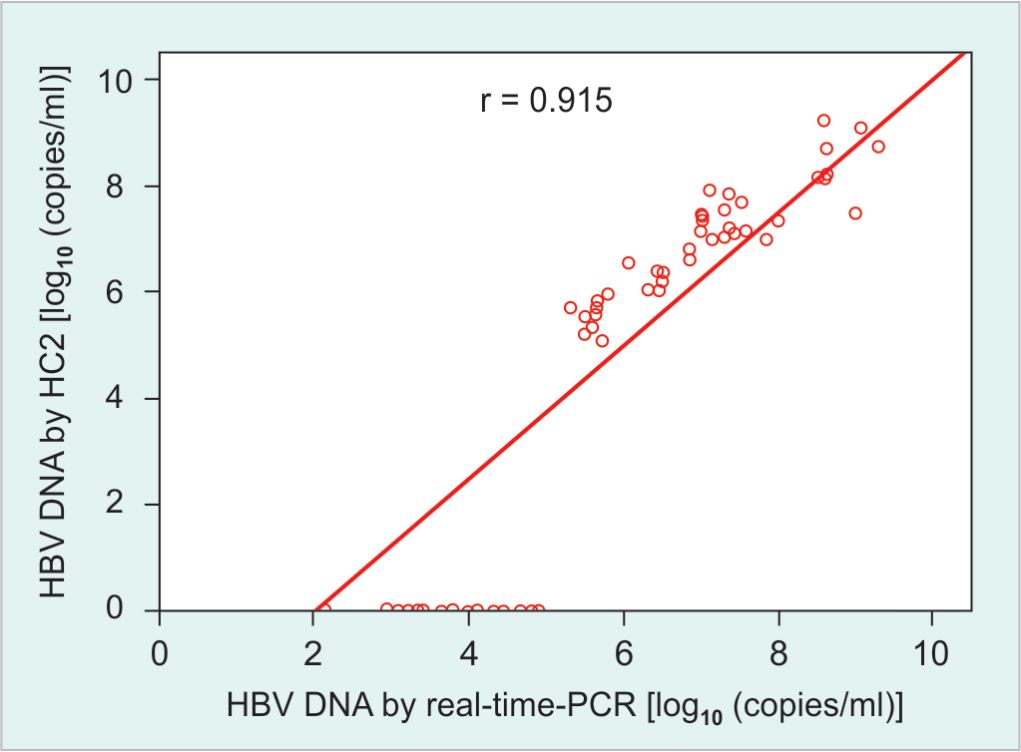
A log-log plot of hepatitis B virus DNA concentrations in 66 samples as measured by real-time-polymerase chain reaction and HC2 assay. Strong correlation (r = 0.915) was observed between results obtained with the two assays for the 40 concordant positive samples. A total of 26 samples tested negative by HC2 assay, but 20 of these had detectable hepatitis B virus DNA by real-time-PCR, while six samples had undetectable DNA by both the assay

**Fig. 2:**
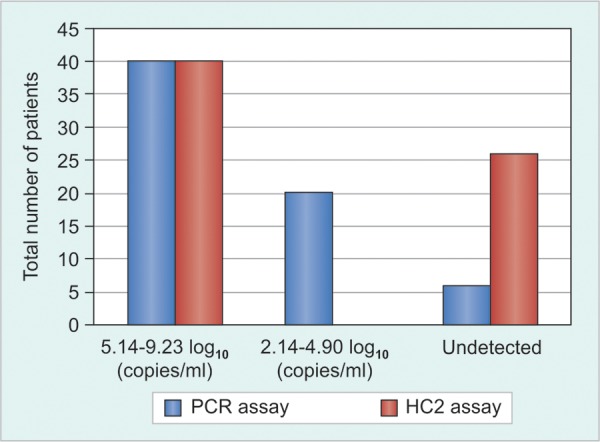
Comparison between real-time-polymerase chain reaction and HC2 assay for HBV DNA detection

Among the total 66 samples that were tested for HBV DNA by both real-time-PCR and HC2 assay in this study, viral load of 40 (60.61%) samples was above 10^5^ copies/ml and their mean viral load was 7.06 ± 1.13 log_10_ copies/ml by real-time-PCR and 6.95 ± 1.08 log_10_ copies/ml by HC2 assay. All 40 samples positive by HC2 also yielded positive results by the real-time-PCR, and the log transformed HBV DNA concentrations obtained with the two assays demonstrated a strong correlation (r = 0.924) (Fig. 1). However, HBV DNA was undetectable in 6 (9.09%) patients by real-time-PCR and in 26 (39.39%) by HC2 assay. The viral load of all the 26 discordant samples from CHB patients that tested negative by HC2 assay was <1.4 × 10^5^ HBV copies/ml. Of these, 20 samples tested positive by real-time-PCR assay. The mean viral load of these samples was 3.67 ± 0.722 [log_10_ (copies/ ml)] and ranged between 2.14 and 4.90 [log_10_ (copies/ml)] (Table 1). Comparison of HBV DNA detection by the two assays is shown in Figure 2.

In the present study, regarding real-time-PCR assay as gold standard, the performance of HC2 assay was compared with real-time-PCR for the detection of HBV DNA. The sensitivity, specificity, positive predictive value (PPV), negative predictive value (NPV) and accuracy were fixed accordingly:

True positive: Out of 66 cases, 40 (60.61%) cases had detectable HBV DNA by both HC2 assay and real-time-PCR, which was regarded as true positive.

True negative: Real-time PCR and HC2 assay both failed to detect HBV DNA in 6 (9.09%) cases and these were considered as true negative.

False negative: The remaining 20 (30.30%) cases had detectable DNA by real-time-PCR, while these were undetectable by HC2 assay and were thus false negative.

False positive: There was no case that had undetectable HBV DNA by real-time-PCR but detected by HC2 assay, which would represent false positive result (Table 2). Thus, performance test of HC2 assay in this study showed a sensitivity of 66%, specificity of 100%, PPV of 100%, NPV of 23% and accuracy of 69%.

Among the 66 patients tested by both real-time-PCR and HC2 assay, 39 (59.09%) were HBeAg positive, while 27 (40.91%) were HBeAg negative. All 39 (100%) HBeAg positive cases had detectable HBV DNA by real-time-PCR, but by HC2 assay HBV DNA was detectable in 29 (74.36%) cases, with mean viral load of 6.37 ± 1.89 [log_10_ (copies/ ml)] and 7.13 ± 1.09 [log _10_ (copies/ml)] respectively. Among HBeAg positive cases, HBV DNA was undetectable in 10 (25.64%) patients by HC2 assay. However, among 27 HBeAg negative cases, 21 (77.78%) had detectable HBV DNA by real-time-PCR and 6 (22.22%) had undetectable DNA. In case of HC2 assay, 11 (40.74%) HBeAg negative cases had detectable and 16 (59.26%) had undetectable DNA. The mean viral load of HBeAg negative cases by real-time-PCR and HC2 was 5.93 ± 1.90 [log_10_ (copies/ml)] and 6.95 ± 1.08 [log_10_ (copies/ml)], respectively (Table 3).

## DISCUSSION

The present study used a highly reproducible and sensitive real-time detection assay based on TaqMan technology for the detection of HBV DNA and compared with commercially available Digene HC2 assay. Among these 66 cases, HBV DNA was detectable in 60 (90.91%) by real-time-PCR and 40 (60.61%) by HC2 assay. Out of these 66 cases, 40 samples which had HBV DNA within the range of HC2 assay described a good correlation (r = 0.915) with real time-PCR (Fig. 1). Studies from Netherlands and UK have reported similar results.^16,17^ HBV DNA was undetectable in 6 (9.09%) patients by real-time-PCR and in 26 (39.39%) by HC2 assay. Of these 26 cases, 20 samples had detectable DNA by real-time-PCR and mean viral load of these samples was 3.67 ± 0.722 [log_10_ (copies/ml)] (Table 1).

According to the performance test, the HC2 assay was found to be an alternative to the real-time-PCR assay. In the study, sensitivity and specificity of HC2 assay were 66 and 100% respectively, where real-time-PCR was regarded as the gold standard (Table 2). Although real-time-PCR assay is a well-accepted and well-practiced method throughout the world,^16^ it is costly and needs very skilled hands from the technical point of view. The main advantage of the real-time-PCR assay is its reproducibility and high degrees of accuracy and precision, which are comparable to those of the HC2 assay.^17^ In contrast, HC2 is cost-effective but more time-consuming. In the present study, seven HBeAg negative cases were undetectable by both real-time-PCR and HC2 assays. These seven cases were further tested for HBsAg and one case was found negative for HBsAg. To fulfill the criteria of the study, this case was excluded.

**Table 1:** **Table 1:** Detection of hepatitis B virus DNA by real-time-polymerase chain reaction and HC2 assay (n = 66)

*Assay type*		*Detectable HBV DNA*				*Undetectable* *HBV DNA*	
		*1.4 × 10^5^ – 1.7 × 10^9^*		*<1.4 × 10^5^ (copies/ml) (copies/ml)*			
Real-time-PCR		40 (60.61%)#		20 (30.30%)		6 (9.09%)	
		7.06 ± 1.13*		3.67 ± 0.72*			
Hybrid capture 2		40 (60.61%)		0		26 (39.39%)	
		6.95 ± 1.08*					

#Figure within parentheses indicates percentage; *HBV DNA (mean ± SD) [log_10_ (copies/ml)]. HBV: Hepatits B virus

**Table 2:** **Table 2:** Comparison of HC2 assay with real-time-polymerase chain reaction for detection of HBV DNA (n = 66)

*Hybrid capture 2 assay for HBV DNA*		*Real-time PCR for HBV DNA*				*Total*	
		*Positive*		*Negative*			
Positive		40		0		40	
Negative		20		6		26	
Total		60		6		66	

Sensitivity: 66%; Specificity: 100%; PPV: Positive predictive value; NPV: 100%; Negative predictive value: 23%; Accuracy: 69%; PCR: Polymerase chain reaction; HBV: Hepatitiis B virus; HC2; Hybrid capture 2

**Table 3:** **Table 3:** Comparison between real-time-PCR assay and HC2 assay for HBV DNA detection (n = 66)

*HBeAg status*		*Number of cases (%)*		*HBV DNA by real-time-PCR*				*HBV DNA by HC2 assay*			
				*Detectable*		*U5detectable*		*Detectable*		*U5detectable*	
Positive		39 (59.09)#		39 (100)		0		29 (74.36)		10 (25.64)
				6.37 ± 1.89*				7.13 ± 1.09*			
Negative		27 (40.91)		21 (77.78)		6 (22.22)		11 (40.74)		16 (59.26)	
				5.09 ± 1.65*				6.46 ± 0.96*			
Total		66		60 (90.91)		6 (9.09)		40 (60.61)		26 (39.39)	
				5.93 ± 1.90*				6.95 ± 1.08*			

#Figure within parentheses indicates percentage; *HBV DNA (mean ± SD) [log_10_ (copies/ml)]. PCR: Polymerase chain reaction; HBV: Hepatitiis B virus; HC2; Hybrid capture 2

Among the 66 cases, 39 (59.09%) cases were positive for HBeAg. Of the HBeAg positive cases, all 39 (100%) and 29 (74.36%) had detectable HBV DNA by PCR and HC2 assay respectively and their viral load was > 105 copies/ml. On the contrary, HBeAg negative cases, were 27 (40.91%) and HC2 assay was able to detect only 11 (40.74%) case but 21 (77.78%) had detectable DNA by real-time-PCR. These patients also had viral load > 10^5^ copies/ml (Table 3). The sensitivity of the HC2 assay might be insufficient for detecting very low HBV DNA levels which is typical in patients undergoing antiviral therapy and those with occult HBV DNA infection.^18^ Therefore, HC2 assay may be used as an alternative to real-time-PCR, usually in CHB patients when HBeAg remain positive.

The present study observed that real-time-PCR is able to detect a wide range of HBV DNA, but HC2 assay could not detect lower levels of viremia. However, comparison of the results of the study observed a good correlation (r = 0.915) between the real-time-PCR and HC2 assay for detection and quantification of HBV DNA. However, this study was limited by lack of genotyping for HBV and determining the frequency of precore/core promoter mutation among HBeAg negative CHB patients. This aspect needs to be evaluated further in future studies with large number of CHB patients.

## CONCLUSION

The present study observed that real-time-PCR had a wide range of detection limit, but HC2 could not detect low level of viremia (<10^5^ copies/ml). However, a good correlation (r = 0.915) was observed between the two assay for the detection and quantification of HBV DNA. Therefore, HC2 assay may be used as an alternative to real-time-PCR in CHB patients who are HBeAg positive.
